# Quantitative evaluation of Gd-EOB-DTPA uptake in focal liver lesions by using T1 mapping: differences between hepatocellular carcinoma, hepatic focal nodular hyperplasia and cavernous hemangioma

**DOI:** 10.18632/oncotarget.18918

**Published:** 2017-07-01

**Authors:** Zhenpeng Peng, Chang Li, Tao Chan, Huasong Cai, Yanji Luo, Zhi Dong, Zi-Ping Li, Shi-Ting Feng

**Affiliations:** ^1^ Department of Radiology, The First Affiliated Hospital, Sun Yat-Sen University, Guangdong, China; ^2^ Department of Diagnostic Radiology, The University of Hong Kong, Pokfulam, Hong Kong

**Keywords:** Gd-EOB-DTPA, MRI, T1 mapping, focal liver lesion, discriminant analysis

## Abstract

**Objectives:**

To investigate the difference of T1 relaxation time on Gd-EOB-DTPA-enhanced MRI in hepatocellular carcinoma (HCC), hepatic focal nodular hyperplasia (FNH) and cavernous hemangioma of liver (CHL), and to quantitatively evaluate the uptake of Gd-EOB-DTPA in these three focal liver lesions (FLLs).

**Results:**

The T1_P_ of CHL was significantly higher than those of HCC and FNH (*P* < 0.05). Reduction of T1 relaxation time on hepatobiliary phase could be observed in all three types of lesions. There were significant differences of T1_P_, T1_E_, T1_D_ and T1_D_% between FNH, CHL and HCC (*P* < 0.001). Spearman correlation analysis revealed that T1_D_% was the best indicator for diagnostic differentiation, with a correlation coefficient of 0.702. Discriminant analysis using three variables (T1_P_, T1_E_, and T1_D_%) showed that the classification accuracy was 88.2%.

**Materials and Methods:**

74 patients diagnosed with focal liver lesions underwent Gd-EOB-DTPA-enhanced MRI including T1 mapping were enrolled, consisting of 51 HCCs, 10 FNHs, and 13 CHLs. T1 relaxation times of these lesions were measured on pre-contrast (T1_P_) and on hepatobiliary phase images at 20 minute after contrast (T1_E_). The reduction of T1 relaxation time on hepatobiliary (T1_D_) and the percentage reduction (T1_D_%) was calculated. The differences of T1_P_, T1_E_, T1_D_ and T1_D_% in these FLLs were analyzed. The usefulness of these parameters for classification of FLLs was evaluated.

**Conclusions:**

Uptake of Gd-EOB-DTPA is different between in HCC, FNH and CHL. These three lesions can be distinguished using T1 mapping.

## INTRODUCTION

Hepatocellular carcinoma (HCC), focal nodular hyperplasia (FNH) and cavernous hemangioma of liver (CHL) are three focal liver lesions (FLLs) commonly encountered in clinical practice. Accurate differentiation of HCC from the latter two benign lesions is of utmost importance since HCC requires early interventions, while FNH and CHL are generally managed conservatively. Radiological examinations, especially magnetic resonance imaging (MRI), has become the method of choice for detection and characterization of FLLs. The differential diagnosis for FLLs on MRI is based on their differences in morphologic features, signal intensity on non-enhanced sequences, and post Gd-DTPA enhancement patterns. However, some atypical lesions such as hypovascular HCCs are still difficult to differentiate from other benign lesions. Furthermore, the existing qualitative assessment methods based on traditional MRI techniques could suffer from interobserver variations, potentially diminishing the accuracy [1–3]. Hepatocyte-specific contrast agent enhanced MRI can provide functional and structural information of FLLs, which greatly improves the diagnostic accuracy of these lesions, especially for lesions showing atypical features on conventional sequences [4].

Gadolinium ethoxybenzyl diethylenetriaminepentaacetic acid or gadoxetic acid (Gd-EOB-DTPA) is hepatocyte-specific contrast agent with a pendant ethoxybenzyl group covalently attached to gadopentetate dimeglumine which can be taken up by hepatocytes via the organic anion transporting polypeptides (OATPs) [4–6]. In humans the contrast agent is absorbed through OATP1B1 and B3 transporters located at the sinusoidal membrane [7]. In patients with normal hepatic and renal function, approximately 50% of gadoxetic acid is excreted by the hepatobiliary system via the multidrug resistance-associated protein (MRP) 2 at the canalicular membrane [8]. In general, the hepatobiliary phase (HBP) images when hepatocytes reach maximum signal intensity, is obtained 10∼20 minutes after contrast administration. The variable contrast uptake by FLLs provides an additional parameter useful for diagnosis in liver imaging. Due to the dual extracellular and hepatobiliary property of Gd-EOB-DTPA, it can provide functional and structural information of the hepatobiliary lesions, in addition to that provided by non-specific gadolinium chelates during the dynamic phases [9–20].

Until recently, most studies concerning Gd-EOB-DTPA-enhanced MRI in FLLs focused on visual assessment of lesion signal intensity and enhancement patterns, or use of the semiquantitative signal to noise ratio (SNR) and contrast to noise ratio (CNR) [17–20]. Kim et al. discovered that optimal CNR is achieved during the hepatobiliary phase in HCCs [21], while another study performed by Gupta et al. found that hemangioma-to-liver CNR peaked in the portal venous phase [22]. Therefore, the comparability of CNR and SNR between lesions of different pathologies may be poor on images produced during any one particular phase. On the contrary, the T1 relaxation time is an intrinsic property of tissues. Changes of T1 values after administration of Gd-EOB-DTPA are directly related to the amount of gadolinium within the lesions, which is in turn related to the differences in vasculature and cellular expression of OATPs and MRP2. Therefore, T1 mapping on Gd-EOB-DTPA-enhanced MRI could be used in quantitative assessment of FLLs and could potentially improve diagnostic accuracy.

In this study, we aim to investigate the difference of T1 relaxation time on Gd-EOB-DTPA-enhanced MRI in HCC, FNH and CHL, and to quantitatively evaluate the uptake of Gd-EOB-DTPA in these three FLLs.

## RESULTS

### Comparison of T1 mapping for different types of FLLs

A total of 74 patients were included in our study. 93 lesions were identified, with the maximum diameter ranging from 7 mm to 120 mm (34.6 ± 31.5 mm). Among these lesions, there were 65 HCCs, 11 FNHs and 17 CHLs.

All the HCCs showed hypointensity on T1 weighted imaging (T1WI) and hyperintensity on T2 weighted imaging (T2WI) relative to adjacent liver parenchyma. After administration of Gd-EOB-DTPA, the HCC lesions revealed heterogeneous enhancement in arterial phase and wash out during portal venous phase. All of the included FNH demonstrated typical imaging features including isointensity to liver on both T1WI and T2WI, homogeneous intense enhancement during the hepatic arterial phase and isointensity or slightly hyperintensity relative to liver in portal venous and delayed phases, with visible central scar. All the CHL showed typical radiological findings such as high signals on T2WI , peripheral globular enhancement or early homogeneous enhancement, fill in phenomenon and prolonged enhancement in the equilibrium phase.

**Figure 1:**
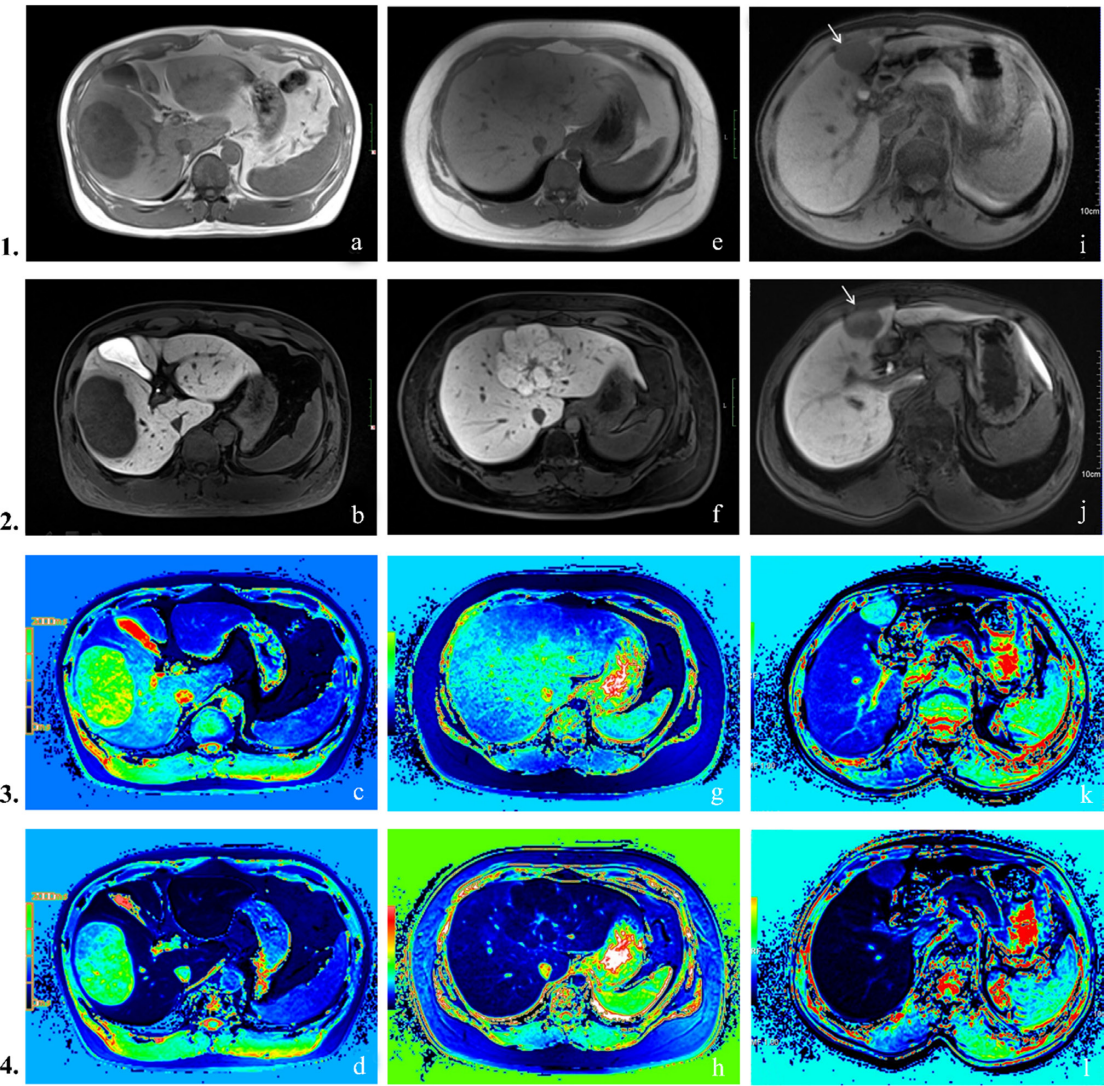
Row 1: pre-contrast T1-weighted images, Row 2: hepatobiliary phase images, Row 3: pre-contrast T1 mapping images, Row 4: hepatobiliary phase T1 mapping images (**A–D**) Hepatocellular carcinoma. Lesion appeared hypointense on both pre-contrast and hepatobiliary phase images. T1_P_ 712 ms, T1_E_ 529 ms, T1_D_ = 183 ms, T1_D_% = 25.7%. (**E–H**) Focal nodular hyperplasia. Characteristic findings include central scar and high signal intensity on the hepatobiliary phase. T1_P_ 648 ms, T1_E_ 89 ms, T1_D_ = 559 ms, T1_D_% = 86.3%. (**I–L**) Cavernous hemangioma of liver. Signal intensities are low in both pre-contrast and hepatobiliary phase T1WI. T1_P_ 1351 ms, T1_E_ 347 ms, T1_D_ = 1004 ms, T1_D_% = 64.3%.

T1_P_ (pre-contrast T1 relaxation time), T1_E_ (hepatobiliary phase T1 relaxation time), T1_D_ (T1 relaxation time reduction) and T1_D_% (T1 relaxation time reduction percentage) were measured and calculated. CHL showed significantly longer T1 relaxation time than the other two lesions on unenhanced MRI, and conspicuous shortening of T1 on hepatobiliary phase. Of all the parameters, T1_D_ was highest in CHL, while FNH had the maximal T1_D_% (Figure 1) (Table 1).

**Table 1:** T1 mapping measurements of FLLs on Gd-EOB-DTPA-enhanced MRI

Lesion	T1_P_(ms)	T1_E_(ms)	T1_D_(ms)	T1_D_%	*N*
**HCC**	1008.6 ± 357.5	629.6 ± 221.0	378.9 ± 258.3	36.5 ± 12.4	65
**FNH**	843.1 ± 286.4	139.7 ± 54.0	703.4 ± 259.3	82.9 ± 6.9	11
**CHL**	1423.0 ± 600.1	500.6 ± 201.6	922.4 ± 424.8	63.9 ± 6.6	17

**Figure 2:**
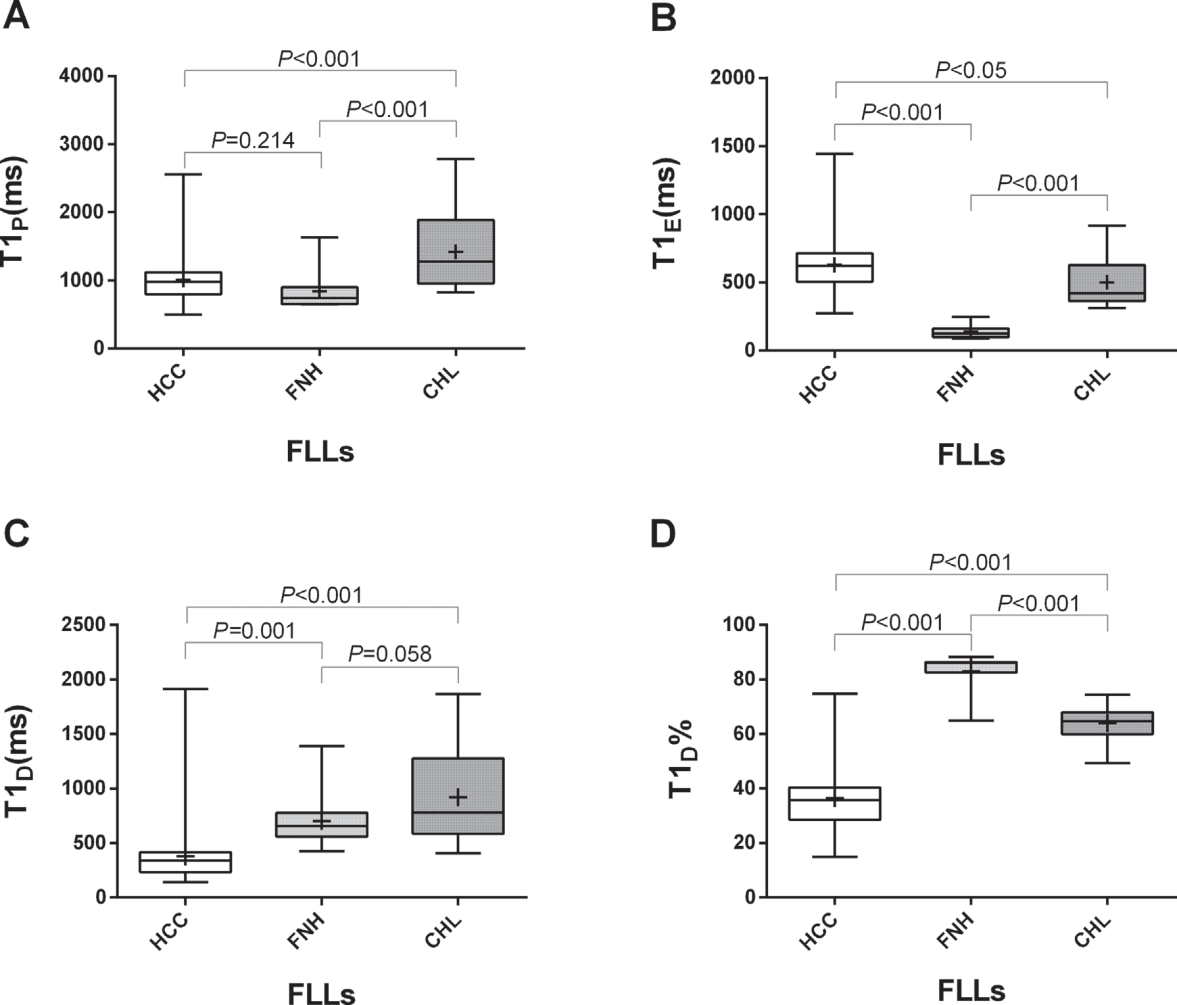
The different values of T1P (**A**), T1_E_ (**B**), T1_D_ (**C**) and T1_D_% (**D**) in HCC, FNH and CHL. Box-and-whisker plots showed that there was statistical significance between each two groups except for T1_P_ between HCC and FNH (*P* = 0.214), and T1_D_ between FNH and CHL (*P* = 0.058).

One-way ANOVA analysis showed that there was statistical significance of T1_P_, T1_E_, T1_D_ and T1_D_% between three groups (*P* < 0.001).Multiple paired comparisons showed no statistical significant difference of T1_P_ between HCC and FNH (*P* = 0.214), and no significant difference of T1_D_ between FNH and CHL (*P* = 0.058). The comparisons of other parameters between each pair of FLLs showed significant difference (Figure 2).

### Correlation analysis

Table 2 shows the correlation between T1 mapping parameters (T1_P_, T1_E_, T1_D_ and T1_D_%) and the three types of lesions by Spearman correlation. T1_D_% showed the best correlation with lesion type, with a correlation coefficient of 0.702.

**Table 2:** Spearman correlation between T1 mapping parameters (T1_P_, T1_E_, T1_D_ and T1_D_%) and the three types of lesions (HCC, FNH and CHL)

	T1_P_	T1_E_	T1_D_	T1_D_%
**Correlation coefficient**	0.179	–0.415	0.665	0.702
***P***	0.086	0.000	0.000	0.000
***N***	93	93	93	93

The threshold value of T1_D_% distinguishes HCC, FNH and CHL: T1 _D_% of HCC was lower than 50%, T1_D_% of FNH was higher than 70%, and 50% < T1_D_ % < 70% was considered to be CHL.

### Linear discriminant analysis

**Figure 3:**
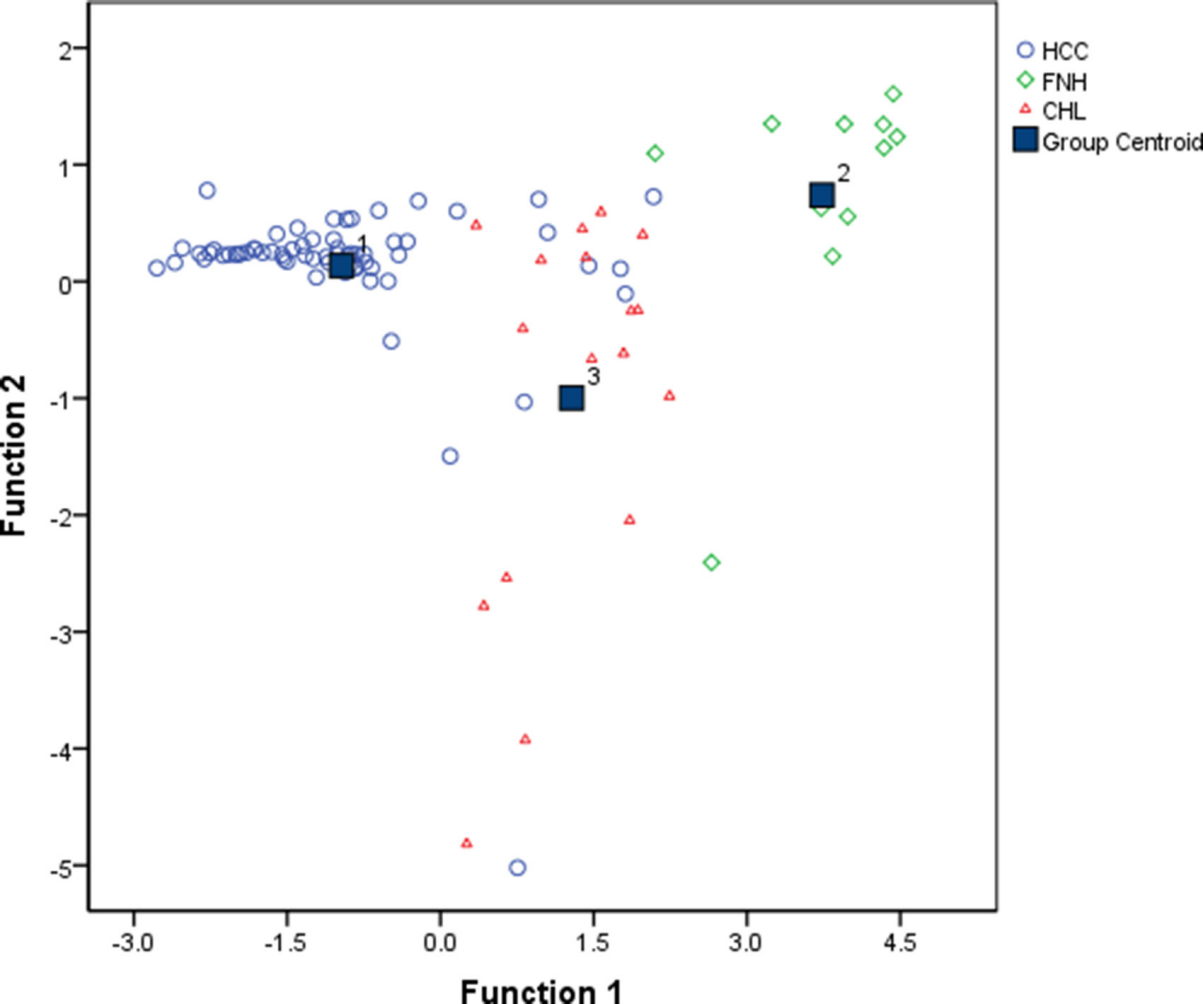
Fisher discriminant analysis plot shows good linear separability of the data points for HCC, FNH and CHL Discriminant function 1 and 2 are linear combinations of the T1 mapping parameters (T1_P_, T1_E_ and T1_D_%). (Function 1 = –5.991 – 0.002*T1_P_ + 0.004*T1_E_ + 0.134*T1_D_%; Function 2 = –1.522 – 0.005*T1_P_ + 0.007*T1_E_ + 0.067*T1_D_%)

Tolerance test demonstrated that T1_D_ is highly correlated with other variables, we therefore ruled out T1_D_ as an explanatory variable of discriminant functions. The coefficients in the Bayesian classification functions defined by the Bayesian discriminant analysis using the three variables (T1_P_, T1_E_ and T1_D_%) are shown in Table 3. Figure 3 showed a good distinction between different groups using the Fisher’s discriminant analysis. From the map we can see that Function 1 accounts for most of the differences between the three groups. Cross validation given in Table 4 estimated that the classification accuracy was 88.2% (error rate = 11.8%).

**Table 3:** Bayesian classification functions

	HCC	FNH	CHL
**Constant**	–41.490	–79.118	–55.440
**T1_P_(ms)**	–0.046	–0.059	–0.045
**T1_E_(ms)**	0.113	0.135	0.114
**T1_D_%**	1.562	2.231	1.787

Classification is determined by calculating discriminant scores from the linear discriminant functions. The highest score determines the type of lesion.

**Table 4:** Classification error from cross validation

Original group	Classification by discriminant analysis			Total	Error rate (%)
	HCC	FNH	CHL		
**HCC**	56	**1**	**8**	65	13.8
**FNH**	0	10	**1**	11	9.1
**CHL**	**1**	0	16	17	5.9
**Total**	57	11	25	93	**11.8**

## DISCUSSION

Our study showed that HCC had the least reduction (T1_D_) and percentage reduction (T1_D_%) of T1 relaxation time in hepatobiliary phase comparing with CHL and FNH, while CHL had a slightly higher T1_D_, and the T1_D_% of FNH was significantly higher than those of CHL and HCC. These findings indicate that T1 mapping combined with Gd-EOB-DTPA-enhanced MRI produces changes of longitudinal relaxation time characteristic of different FLLs, which hence provides quantitative information for distinguishing between the FLLs. In our study, T1_D_% was found to be the best parameter in characterization. Unlike T1_P_, T1_E_ or T1_D_, T1_D_% is independent of varying imaging parameters, which avoids errors caused by using different MRI platforms, or other technical factors. We have previously found T1_D_% to be an effective predictor for the differentiation of different degrees of HCC in an earlier study [47]. This study was the first to provide the threshold values of T1_D_% for distinguishing between HCC, FNH and CHL: T1 _D_ % of HCC was the lowest (< 50% ), T1_D_% of FNH was the highest (> 70%) , and T1_D_% from 50% to 70% was likely to be CHL.

The mechanisms of hepatocyte-specific contrast agent Gd-EOB-DTPA uptake and excretion have been elaborated in many previous studies [4–8]. It has a higher protein binding capacity than the extracellular contrast Gd-DTPA, thus increasing the T1 relaxivity and signal enhancement in blood and liver relative to Gd-DTPA, which explains the lower clinical recommended dose of Gd-EOB-DTPA (0.025 mmol/kg vs. 0.1 mmol/kg for Gd-DTPA) [23, 24]. The unique properties of Gd-EOB-DTPA add substantial information including both qualitative and quantitative assessment during the hepatobiliary phase, improving the detection and characterization of focal liver lesions [25–29]. On the other hand, because of increased parenchymal enhancement during the equilibrium phase in dynamic contrast-enhanced MRI, the lesion-to-liver contrast may be decreased relative to traditional extracellular contrast-enhanced MRI, making the delayed enhancement less useful for diagnosing lesions such as hemangioma and cholangiocarcinoma [22]. In order to make an accurate diagnosis, we need more quantitative information. Unlike signal intensity, which may be affected by many factors such as radiofrequency amplifier, T1 relaxation time is an intrinsic property of tissues. It has been illustrated that a change in the longitudinal relaxation rate (1/T1) is directly proportional to the concentration of contrast agent gadolinium in the tissues [30]. Therefore, we applied T1mapping technique in this study to quantitatively evaluate the characteristics of different FLLs. In the recent years, use of T1 mapping techniques have been simplified and become more readily integrated into clinical MRI examination. Pre-contrast T1 and post-contrast T1 maps can be acquired automatically during MRI scan. Some recent studies have reported that Gd-EOB-DTPA enhanced MRI combined with T1 mapping can be used to estimate liver function [31] and distinguish hepatic metastasis from heamangioma [32]. However, few studies focused on the application in differential diagnosis of HCC, FNH and CHL. In our study, we applied Syngo MapIt, a software using 3D-VIBE that enables fast imaging [32], to evaluate the usefulness of T1 mapping in the differentiation of HCC, FNH, and CHL.

On dynamic contrast enhanced MRI, HCC typically shows evident homogeneous or heterogeneous enhancement in arterial phase and relatively reduced enhancement in portal venous phase. Since most HCCs do not selectively take up Gd-EOB-DTPA, they appear hypointense relative to liver parenchyma which is markedly enhanced on hepatobiliary phase [33]. Our study found that HCC had the least reduction (T1_D_) or percentage reduction (T1_D_%) of T1 relaxation time in hepatobiliary phase among all three lesions, which could be explained by the least concentration of Gd-EOB-DTPA retained in the foci. Narita et al. reported that some highly-differentiated HCCs with expression of OATP1B3 might take up Gd-EOB-DTPA during HBP [34], accounting for the low level of reduction in T1 values (mean value 36.5%).

On traditional Gd-DTPA-enhanced MRI, CHL typically shows peripheral nodular enhancement in arterial phase which progresses centripetally in portal venous phase and tends to retain contrast in delayed phase. However, with Gd-EOB-DTPA, CHLs may not be completely filled with contrast agent in the portal venous or equilibrium phases due to hepatocyte uptake reducing the amount of contrast medium in the blood [35]. Since CHLs contain no functioning hepatocytes they are hypointense relative to the highly enhanced parenchyma during the hepatobiliary phase [22]. This phenomenon is called “pseudo washout” sign [36]. In the present study, the precontrast T1 relaxation time (T1_P_) of CHL was significantly longer than those of HCC or FNH. This could be explained by the abundant amount of blood inside the dilated sinusoids, lengthening the longitudinal relaxation time. Previous studies have figured out that in HBP a part of gadoxetic acid distributes in extracellular space pools in the dilated sinusoidal spaces in hepatic hemangioma [32], indicating the possibility of prominent T1-shortening effect during HBP, which is compatible with our results.

Histologically, FNH is composed of lobules of normal-appearing hepatocytes with blind-ending biliary ductules [37]. On dynamic extracellular contrast-enhanced MRI, FNH typically appears hyperintense in arterial phase and iso- or hypointense in portal venous and delayed phase. The OATP1B1/B3 expression in FNH is increased while the expression of MRP2 is similar to that of the normal liver [38]. The hepatocytes inside FNH are typically increased in density compared to normal liver parenchyma [10]. As a result, FNH usually presents iso- to hyperintense relative to liver parenchyma during the hepatobiliary phase [39]. It is noticeable that although CHL might have a slightly higher T1_D_, the percentage reduction T1 values (T1_D_%, mean value 82.9%) of FNH was significantly higher than those of CHL and HCC.

Multiple comparisons of T1 mapping between HCC, FNH and CHL found that precontrast T1 relaxation times (T1_P_) of HCC and FNH had no significant differences, suggesting that precontrast T1 relaxation time alone is insufficient for accurate diagnosis. Although the T1 reduction level (T1_D_) of CHL was slightly higher than that of FNH, they showed no statistical significance, excluding the T1_D_ measurements as useful parameter for differential diagnosis between the two lesions. Spearman correlation analysis demonstrated that the correlation coefficient of T1_D_% is highest among all parameters, indicating that T1_D_% may be a promising parameter for differentiation of HCC, FNH, and CHL.

Compared to traditional MRI, the advantages of using Gd-EOB-DTPA enhanced MRI combined with T1 mapping is to facilitate quantitative evaluation of Gd-EOB-DTPA uptake in the lesion, in addition to the dynamic enhancement patterns of these FLLs available in both techniques. Therefore, discriminant function could be built up to distinguish the three nodules using the three variables (T1_P_, T1_E_ and T1_D_%). Our study showed that Function 1 accounts for most of the differences between the three groups, which indicates a high diagnostic efficacy and makes a good distinction between different FLLs. Furthermore, cross validation showed that the discriminant analysis based on three variables (T1_P_, T1_E_ and T1_D_%) increased the accuracy of classification to 88.2%, compared to the analyses with single variable. So far as we know, discriminant function by using T1 mapping parameters has not been reported previously. Based on our study, Gd-EOB-DTPA enhanced MRI combined with T1 mapping is considered to improve the diagnostic accuracy of FLLs, especially for nodules without typical imaging features. Thus, assessment of T1 map data (T1_P_, T1_E_, T1_D_ and T1_D_%) is justified, even if it increases the post-processing workload. It is anticipated that future developments in automatic diagnosis of focal liver lesions based on the data would render the task efficient enough for clinical practice.

The present study has some limitations. Firstly, there were only three types of focal liver lesions included in the study. Secondly, the numbers of FNH and CHL were small, and all the FNH and CHL were diagnosed by typical imaging features. Therefore, FNH and CHL without typical imaging features might be missed in the study. These pitfalls could potentially contribute to bias and affect applicability of the results. Thirdly, previous studies revealed that there existed some correlation between relative HBP signal intensity or enhancement patterns and grade of HCC [40–46]. However, the degree of differentiation in HCC was not analyzed in this study.

In conclusion, quantitative evaluation of Gd-EOB-DTPA uptake in FLLs using T1 mapping is feasible. T1 mapping in Gd-EOB-DTPA-enhanced MRI reflects changes of longitudinal relaxation time of focal liver lesions, which is proportional to the concentration of Gd-EOB-DTPA and provides quantitative information for lesion characterization. The percentage reduction T1 relaxation time in hepatobiliary phase combined with discriminant analysis has excellent sensitivity and specificity in the differential diagnosis of HCC, FNH, and CHL. Therefore, T1 mapping is a promising quantitative method in focal liver lesion diagnosis.

## MATERIALS AND METHODS

### Patients

This was a retrospective study. The study was conducted in accordance with ethical guidelines for human research and was compliant with the Health Insurance Portability and Accountability Act (HIPAA). As such, the study received IRB or ethical committee approval, and the requirement for informed consent was waived.

Between July 2012 and February 2015, 74 patients (51 men, 23 women) with an age range of 21–89 years (40.3 ± 11.5 years) were enrolled. There were 51 patients diagnosed with 65 HCC, 10 patients diagnosed with 11 FNH, and 13 patients diagnosed with 17 CHL. All of the HCCs were confirmed by surgery or biopsy. FNH and CHL were diagnosed on the basis of typical MRI features. FNH was diagnosed when all of the following findings were identified in the lesion: (1) isointense or slightly hypointense compared with the liver on T1WI. (2) isointense or slightly hyperintense on T2WI. (3) homogeneous intense enhancement during the hepatic arterial phase. (4) isointense or slightly hyperintense in relation to the adjacent liver parenchyma during hepatic venous and delayed phases.(5) a visible central scar seen as a hyperintense focus on T2-weighted images and as hypointense on unenhanced T1-weighted images, with some contrast material uptake during the delayed phase [47]. CHL was diagnosed when the typical radiological findings were identified in the lesions, such as high signal intensity compared with the liver on T2WI , peripheral globular enhancement, early total enhancement, presence of the fill in phenomenon and prolonged enhancement in the equilibrium phase [32].

### MRI protocol

All MRI examinations were performed on a clinical 3.0-T scanner (Magnetom Verio, Siemens Healthcare Sector, Erlangen, Germany). The body array coil (3T; 8-channel body matrix coil) was used in all examinations. All patients fasted for 6∼8 hours prior to examination and were trained for breath holding. Bellyband was used during examination.

All patients received a body weight adjusted dose of Gd-EOB-DTPA (Primovist^®^, 0.1 ml/kg body weight) administered as bolus injection with a flow rate of 1 ml/s, flushed with 30 ml of normal saline with a flow rate of 2 ml/s.

The conventional imaging sequence included axial in- and out-of-phase, T1WI, and T2WI. For T1 mapping, a dual flip angle 3D gradient echo sequence with volumetric interpolated breath-hold examination (VIBE) was performed before and 20 minutes after injection of Gd-EOB-DTPA consistently in each patient (Table 5). Each T1 mapping sequence took 20.15s.

**Table 5:** Acquisition parameters of the MRI protocol (FA = flip angle, VIBE = volumetric interpolated breath-hold examination, HBP = hepatobiliary phase)

	TR (ms)	TE (ms)	FA	Time (s)	Slice Thickness (mm)	Matrix	FOV
**Pre-contrast**							
**T1WI**	225	2.2	70	19.42	6	200 × 320	258 × 330
**in- and out-of-phase**	200	2.5,3.7	65	17.67	6	154 × 256	248 × 330
**T2WI**	1600	91	150	0.84	3	410 × 512	350 × 350
**T1 mapping (before)**	4.4	1.2	2, 12	20.15	2	154 × 256	248 × 330
**VIBE**	3.3	1.2	13	8.21	2	96 × 256	248 × 330
**HBP**							
**T1WI**	225	2.2	70	19.42	6	200 × 320	258 × 330
**T1 mapping (after)**	4.4	1.2	2, 12	20.15	2	154 × 256	248 × 330

### Image analysis

The T1 maps of the liver were generated with the evaluation tool for calculating T1 relaxation times (Siemens Leonardo Syngo 2009B). T1 relaxation times on T1 mapping images were measured before and 20 minutes after the administration of the contrast medium (recorded as T1_P_ and T1_E_ respectively). Regions of interest (ROIs) were drawn in the most homogeneous appearing portion of the lesion, avoiding tumor capsule, necrosis, fat, vessels, hemorrhage or central scar. Round ROIs were drawn as large as possible within the boundary of the lesion. The sizes of ROI were within the range of 4∼10 mm^2^, and these ROIs were identical in size and position on corresponding slices before and after contrast.

Two experienced radiologists drew the ROIs independently, and would reach a consensus after discussion if there were any conflicts. Each lesion was measured 3 times and mean value was applied for calculating reduction of T1 relaxation times (T1_D_) after enhancement and its percentage reduction (T1_D_%) as follows [47]:

$$\begin{array}{l} \text{T}1_{\text{D}}=\text{T}1_{\text{P}}-\text{T}1_{\text{E}}\\ \text{T}1_{\text{D}}\%=\left[\left(\text{T}1_{\text{P}}\text{-T}1_{\text{E}}\right)/\text{T}1_{\text{P}}\right]\times 100\%\; \end{array}$$

T1 maps were also color-coded using a visualization tool of the imaging software OsiriX.

### Statistical analysis

All statistical analyses were done using SAS (version 9.4, SAS Institute, Cary, NC). One-way analysis of variance (ANOVA) and multiple comparisons between groups were used to analyze differences of these values between HCC, FNH and CHL. Spearman correlation was also done to analyze the correlation between T1 mapping parameters and the three types of lesions. All tests were two-sided and *p* value < 0.05 indicated significant difference.

The ability of T1 mapping to classify individual images of patients with focal liver lesions into the correct group was evaluated using linear discriminant analysis (LDA), a statistical method used in machine learning to determine the linear combination of variables best able to classify a given set of data. Fisher’s and Bayesian discriminant analysis were performed separately. Classification functions were determined by the linear combination of explanatory variables which maximized the separation between groups. Cross validation was performed by jackknife or leave-one-out method to assess the accuracy of prediction with different classification rules. Classification accuracy was defined as the ratio between the number of cases correctly classified and the total number of cases.
